# Differences of inter-tract correlations between neonates and children around puberty: a study based on microstructural measurements with DTI

**DOI:** 10.3389/fnhum.2013.00721

**Published:** 2013-10-29

**Authors:** Virendra Mishra, Hua Cheng, Gaolang Gong, Yong He, Qi Dong, Hao Huang

**Affiliations:** ^1^Advanced Imaging Research Center, University of Texas Southwestern Medical CenterDallas, TX, USA; ^2^Department of Radiology, Beijing Children's HospitalBeijing, China; ^3^Department of Radiology, Capital Medical UniversityBeijing, China; ^4^State Key Laboratory of Cognitive Neuroscience and Learning & IDG/McGovern Institute for Brain Research, Beijing Normal UniversityBeijing, China; ^5^Department of Radiology, University of Texas Southwestern Medical CenterDallas, TX, USA

**Keywords:** brain development, neonate, DTI, microstructure, inter-tract correlation, homologous

## Abstract

The human brain development is a complicated yet well-organized process. Metrics derived from diffusion tensor imaging (DTI), including fractional anisotropy (FA), radial (RD), axial (AxD), and mean diffusivity (MD), have been used to noninvasively access the microstructural development of human brain white matter (WM). At birth, most of the major WM tracts are apparent but in a relatively disorganized pattern. Brain maturation is a process of establishing an organized pattern of these major WM tracts. However, how the linkage pattern of major WM tracts changes during development remains unclear. In this study, DTI data of 26 neonates and 28 children around puberty were acquired. 10 major WM tracts, representing four major tract groups involved in distinctive brain functions, were traced with DTI tractography for all 54 subjects. With the 10 by 10 correlation matrices constructed with Spearman's pairwise inter-tract correlations and based on tract-level measurements of FA, RD, AxD, and MD of both age groups, we assessed if the inter-tract correlations become stronger from birth to puberty. In addition, hierarchical clustering was performed based on the pairwise correlations of WM tracts to reveal the clustering pattern for each age group and pattern shift from birth to puberty. Stronger and enhanced microstructural inter-tract correlations were found during development from birth to puberty. The linkage patterns of two age groups differ due to brain development. These changes of microstructural correlations from birth to puberty suggest inhomogeneous but organized myelination processes which cause the reshuffled inter-tract correlation pattern and make homologous tracts tightly clustered. It opens a new window to study WM tract development and can be potentially used to investigate atypical brain development due to neurological or psychiatric disorders.

## Introduction

The human brain is complicated yet well organized. The major cerebral white matter (WM) tracts connecting different brain regions are involved in different brain functions. These major cerebral WM tracts are often categorized into different tract groups based on their distinct functions. There are roughly four tract groups, namely limbic, projection, callosal, and association tract groups (e.g., Wakana et al., 2004; Huang et al., 2012a,b), for cerebral WM tracts. The WM tracts within a tract group perform similar functions. For example, limbic tracts underlie the connectivity in the limbic system and association tracts connect between cerebral cortical areas. The pair of tracts in both cerebral hemispheres belongs to the same tract group and is considered as homologous tracts. At birth, most of major WM tracts are well formed (e.g., Huang et al., 2006; Oishi et al., 2011), except the arcuate fasciculus which is a part of the superior longitudinal fasciculus (SLF) and related to language function.

The water molecules all over the human brain tend to diffuse more freely along the WM fiber bundle, instead of perpendicular to it. This diffusion property can be measured noninvasively with diffusion MRI (dMRI), a modality of MRI. The widely used diffusion tensor imaging (DTI) (Basser et al., 1994) characterizes the water diffusion properties in the brain voxels with a tensor model. Fractional anisotropy (FA) (Pierpaoli and Basser, 1996; Beaulieu, 2002) and mean diffusivity (MD), derived from DTI, have been widely used to quantify the microstructural properties of the WM voxels. Other than FA or MD, the other two DTI-derived metrics, radial diffusivity (RD) and axial diffusivity (AxD), convey unique information related to myelination and axonal integrity, respectively (Song et al., 2002). The four DTI-derived metrics, FA, RD, AxD, and MD, characterize different aspects of diffusion tensor and are highly sensitive to WM microstructural changes.

Compared to voxel-based morphometry (VBM), recent tract analyses (Yushkevich et al., 2008; Goodlett et al., 2009; O'Donnell et al., 2009; Zhang et al., 2010; Colby et al., 2012) including ours (Huang et al., 2011, 2012a,b) have become important due to great functional and clinical significance of the tracts. These WM tracts can be noninvasively traced with tractography based on diffusion MRI (dMRI) (e.g., Conturo et al., 1999; Jones et al., 1999a; Mori et al., 1999; Basser et al., 2000; Stieltjes et al., 2001; Catani et al., 2002; Parker et al., 2002; Lazar et al., 2003; Behrens et al., 2007). And the heterogeneous WM tracts can be noninvasively segmented with the traced fibers. With these segmented WM tracts as binary masks for the maps of DTI-derived metrics, the microstructural properties of the WM tracts can be quantified.

Dramatic microstructural changes take place during normal human brain development from birth to puberty, which are two landmark time points in early brain development. Birth marks the beginning time point of postnatal brain development. Puberty marks the end of the child development and beginning of adolescence. The four DTI derived metrics, FA, RD, AxD and MD, have been incorporated in numerous studies investigating WM microstructural changes for infants, children and adolescents during development. The human brain development process is usually characterized with significant increases in FA (e.g., Barnea-Goraly et al., 2005; Snook et al., 2005; Eluvathingal et al., 2007; Dubois et al., 2008; Lebel et al., 2008; Gao et al., 2009; Giorgio et al., 2010; Schmithorst and Yuan, 2010; Tamnes et al., 2010; Westlye et al., 2010) and significant decreases in MD, AxD and RD (e.g., Snook et al., 2005; Eluvathingal et al., 2007; Dubois et al., 2008; Lebel et al., 2008; Gao et al., 2009; Giorgio et al., 2010; Schmithorst and Yuan, 2010; Tamnes et al., 2010; Westlye et al., 2010). On the other hand, it was shown with DTI-derived metrics of a cohort of adults that specific WM tracts involved in similar functions vary in a similar pattern with each other across different individuals (Wahl et al., 2010; Li et al., 2012), while hemispheric asymmetries of DTI-derived metrics in homologous pairs of WM tracts (Bonekamp et al., 2007; Wilde et al., 2009) have been reported. However, from perspective of brain development, whether or not the significant inter-tract correlations exist at birth and around puberty is still unclear. Furthermore, it remains elusive if these inter-tract correlations will be strengthened and how the correlation patterns change from birth to puberty.

During the development from birth to puberty, the human brain is likely to change from a more randomized state to a more balanced and organized state. In this study, we hypothesized that inter-tract correlations become stronger and the correlation patterns are reshuffled from birth to puberty. Specifically, the reshuffling process will cause more homologous tracts to form tight relationship. DTI data were acquired from 26 normal neonates and 28 normal children around puberty. The following 10 major WM tracts covering limbic, association, commissural and projection tract groups were selected for tract-level measurements of DTI metrics of each subject: left and right corticospinal tract (CST_L and CST_R), left and right inferior fronto-occipital fasciculus (IFO_L and IFO_R), left and right cingulate part of cingulum tract (CGC_L and CGC_R), left and right hippocampal part of cingulum tract (CGH_L and CGH_R), forceps major (FMajor) and forceps minor (FMinor). The tract level comparisons of all four DTI-derived metrics were conducted between the two age groups. Spearman's pairwise inter-tract correlations were performed. We tested if significant correlations of homologous WM tracts exist in neonates and children around puberty. After obtaining four 10 by 10 inter-tract correlation matrices corresponding to four DTI-derived metrics, FA, RD, AxD, and MD, for each age group, we tested these correlation matrices against the identity matrix or a matrix with equal non-diagonal entries. We then assessed if the inter-tract correlations become statistically stronger from birth to puberty. In addition, hierarchical clustering was performed with the pairwise correlations based on FA, RD, AxD, and MD measurements for each age group to reveal the pattern of clustering in either age group and reveal the pattern shift from birth to puberty.

## Materials and methods

### Subjects and data acquisition

Twenty six normal neonates (14 males; age: 37 to 43 gestational weeks with mean and standard deviation 40.1 ± 2.0 gestational weeks) and 28 normal children around puberty (15 males; age: 9.5–15 years with mean and standard deviation 12.0 ± 2.3 years), free of current and past neurological or psychiatric disorders, were recruited at Children's Medical Center (CMC) at Dallas and Advanced Imaging Research Center (AIRC) of the University of Texas Southwestern Medical Center (UTSW), respectively. The parents of all the subjects gave written informed consents approved by Institutional Review Board of UTSW.

Two 3T Philips Achieva MR systems at CMC and AIRC were used to acquire dMRI of neonate and child group, respectively. dMRI data were acquired using a single shot echo planar imaging (EPI) with SENSE parallel imaging scheme (SENsitivity Encoding, reduction factor = 2.3). dMRI parameters for neonates were: *FOV* = 200/200/100 mm, in-plane imaging matrix = 100 × 100, axial slice thickness = 2 mm. dMRI parameters for children around puberty were: *FOV* = 224/224/143 mm, in-plane imaging matrix = 112 × 112, axial slice thickness = 2.2 mm. The common parameters for dMRI acquisition of both neonate and child group were: *b*-value = 1000 s/mm^2^, *TE* = 97 ms, *TR* = 7.6 s, 30 independent diffusion-weighted directions (Jones et al., 1999b) and 2 repetitions to increase signal-to-noise ratio (SNR).

### DTI preprocessing

dMRI acquired from all the subjects was processed offline using DTIStudio (mristudio.org; Jiang et al., 2006). dMRI images for each subject were corrected for motion and eddy current by registering all the diffusion weighted images to the b0 image using a 12-parameter (affine) linear image registration with automated image registration (AIR) algorithm (Woods et al., 1998). After the registration, six independent elements of the 3 × 3 diffusion tensor (Basser et al., 1994) were determined by multivariate least-square fitting of diffusion weighted images. The tensor was diagonalized to obtain three eigenvalues (λ_1 − 3_) and eigenvectors (ν_1 − 3_). FA, MD, AxD and RD, derived from DTI, were obtained for all the subjects with the following equations of eigenvalues:

$$\begin{array}{l} \;FA=\frac{\sqrt{{\left(\lambda_{1}-\lambda_{2}\right)}^{2}+{\left(\lambda_{1}-\lambda_{3}\right)}^{2}+{\left(\lambda_{2}-\lambda_{3}\right)}^{2}}}{\sqrt{2}\sqrt{\lambda_{1}^{2}+\lambda_{2}^{2}+\lambda_{3}^{2}}}\\ \;MD=(\lambda_{1}+\lambda_{2}+\lambda_{3})/3\\ AxD=\lambda_{1}\\ \;RD=(\lambda_{2}+\lambda_{3})/2 \end{array}$$

### Tract-level measurements of DTI metrics

The following 10 major WM tracts were selected for tract-level measurements of DTI metrics, left and right corticospinal tract (CST_L and CST_R), left and right inferior fronto-occipital fasciculus (IFO_L and IFO_R), left and right cingulate part of cingulum tract (CGC_L and CGC_R), left and right hippocampal part of cingulum tract (CGH_L and CGH_R), forceps major (FMajor) and forceps minor (FMinor). These tracts could be reproducibly traced with DTI of all neonates and children and cover all four major tract groups, namely projection, limbic, commissural and association tract group. Other major WM tracts such as SLF could not be traced reproducibly with our cohort of neonate DTI dataset. Following the literature (Wakana et al., 2007), the tractography protocol described in details below was used to trace all these tracts. DTIstudio (mristudio.org) was used to conduct the tractography. The binary masks of the individually traced tracts were used to compute the tract-level FA, RD, AxD, and MD. The test-retest reliability was quantified by coefficient of variation (CV) and κ values of variability shown in Supplemental Table 1, after tracing the tracts below 3 times with the data from 3 subjects randomly selected from each group. All CV values are less than 2% and κ values are greater than 95% for both neonate an child group, indicating almost perfect test-retest reliability and almost perfect agreement of measurements among different tests in both neonate and child group.

#### CST-L and CST-R

For the first ROI, the entire cerebral peduncle of the desired hemisphere was delineated at the level of the decussation of the superior cerebellar peduncle using an axial slice. “OR” operation was used to select all the CST fibers in this hemisphere that reach the primary motor cortex. The second ROI was then drawn at the most ventral axial slice that identifies the cleavage of the central sulcus. “AND” operation is performed at this axial slice to select all the CST fibers in this hemisphere. The fibers running through to the opposite hemisphere were removed using the “NOT” operation.

#### CGC-L and CGC-R

For the first ROI, a coronal plane was selected at the middle of the splenium of the corpus callosum (CC) using the mid-sagittal plane and the region containing the entire cingulum in the desired hemisphere is selected. All the fibers in this coronal plane passing through the cingulum were selected using the “OR” operation. The second ROI was drawn by selecting a coronal plane in the middle of the genu of the CC and all the CGC fibers were selected using the “AND” operation.

#### CGH-L and CGH-R

For the first ROI, a coronal plane in the middle of the splenium of the CC was selected using the mid-sagittal plane and the cingulum below the CC of the desired hemisphere was delineated. All the fibers in this coronal plane passing through the cingulum were selected using the “OR” operation. The second ROI was drawn at a coronal slice anterior to the pons using the mid-sagittal plane and the fibers passing through the cingulum in this hemisphere were selected using the “AND” operation.

#### IFO-L and IFO-R

For the first ROI, a coronal slice at the middle point between the posterior edge of the cingulum and the posterior edge of the parieto-occipital sulcus was selected and the entire occipital lobe of the desired hemisphere was delineated. All the fibers in this hemisphere were selected using the “OR” operation. The second ROI was drawn at the anterior edge of the CC using a coronal slice and all the fibers in this hemisphere were selected using the “AND” operation. The fibers running through to the thalamus were removed by using the “NOT” operation.

#### FMajor

For the first ROI, a coronal plane including only the left occipital lobe was selected at the most posterior edge of the parieto-occipital sulcus. The “OR” operation in this coronal plane delineated all the fibers of FMajor. The second ROI was drawn in the same coronal plane on the right hemisphere using the “AND” operation such that all the fibers in the right occipital lobe was selected.

#### FMinor

For the first ROI, a coronal plane at the middle point between the anterior tip of the frontal lobe and the anterior edge of the genu of the CC was selected using the mid-sagittal plane. The “OR” operation was used to select all the fibers in the entire left hemisphere. The second ROI was drawn in the same coronal plane on the right hemisphere and all the fibers in the right hemisphere were selected using the “AND” operation.

### Inter-tract correlation analysis

Shapiro-Wilk normality test was performed with DTI-derived metrics of all the 10 WM tracts of 26 neonate brains and 28 child brains. Distributions of DTI-derived metrics for most of the tracts in the neonate group did not show significant difference (*p* > 0.05) from normality. However, distributions of DTI-derived metrics of most of the tracts in the child group differed significantly (*p* < 0.05) from normality. Hence, following the method in the literature (Wahl et al., 2010), non-parametric Spearman's rank correlation coefficient ρ was used to measure all correlations. Subsequently, a correlation matrix was constructed for each of the 4 DTI-derived metrics using pairwise correlation values between any two tracts. Symmetric correlation matrices were obtained with a value of unity along the diagonal of the correlation matrix. The diagonal element represents perfect correlation of the DTI-derived parameter of the tract with itself and the off-diagonal element represents the correlation of the DTI derived parameter of one tract with that of another tract. There were 10^*^(10−1)/2 = 45 nontrivial independent correlation values in each correlation matrix.

### Statistical analysis

Two independent tests of correlation matrices were performed to evaluate if the correlation matrices were significantly different from identity and homogeneous matrix (Rencher, 2002; Wahl et al., 2010). The null hypothesis to test for identity was the correlation matrix is an identity matrix, $\Pi_{0}=\left(\begin{array}{ccc} 1 & 0 & 0\\ \vdots & \ddots & \vdots \\ 0 & \cdots & 1 \end{array}\right)$. The size of the correlation matrix was 10 × 10 as we were testing for pairwise correlations of 10 independent tracts. To test the correlation matrices for homogeneity, the null hypothesis was that the correlation matrix was homogeneous and that the non-diagonal elements of the matrix were equal, i.e.,: $\mu_{0}=\left(\begin{array}{ccc} 1 & \rho & \rho \\ \vdots & \ddots & \vdots \\ \rho & \cdots & 1 \end{array}\right)$. This null hypothesized homogeneous correlation matrix was derived by following the procedure outlined in the literature (Rencher, 2002; Wahl et al., 2010). Bonferroni correction was conducted for both test of correlation matrix against identify matrix and test of correlation matrix against matrix with equal non-diagonal elements. Once the DTI-derived correlation matrices were found to be significantly different from identity and homogeneity within each group, correlation matrices of each DTI-derived metric were compared between the two age groups. Spearman's rank correlation coefficients were converted to *z* values by using Fisher's r-to-z-transform (Fisher, 1915). Note that *r* in Fisher's r-to-z-transform is the spearman's rank correlation coefficient ρ in this study. *Z*-statistics was then performed to identify the pair of tracts that showed significant change in correlation strength from birth to puberty.

### Hierarchical clustering analysis

Hierarchical clustering methods were used to characterize the patterns of inter-tract correlation in each matrix among the groups. We used 1-ρ, where ρ is the Spearman's rank correlation coefficient, as a measure of distance or dissimilarity between the WM tracts for the purpose of clustering. Hierarchical clustering was performed using hclust function in R version 2.15.2. Depending on the correlation coefficient, different WM tracts were successively grouped into larger groups and the results were visualized as a dendrogram. WM tracts that had stronger correlation among themselves were fused together and were linked together. To characterize the uncertainty of the linkage among WM tracts and reduce the standard error in the percentage of confidence level for each cluster, a multi-scale bootstrap with 1000 repetitions of the analysis was performed using pvclust function in R (Suzuki and Shimodaira, 2006). The multi-scale bootstrap analysis yields an approximately unbiased *p*-value of each linkage in hierarchical clustering and has been applied in various other studies (Shimodaira, 2002, 2004; Wahl et al., 2010). The threshold for determining statistical significance for the grouping of tracts was set at an unbiased *p*-value of 0.05 or 95% confidence interval.

## Results

### Changes of white matter microstructure from neonates to children around puberty

**Figure 1:**
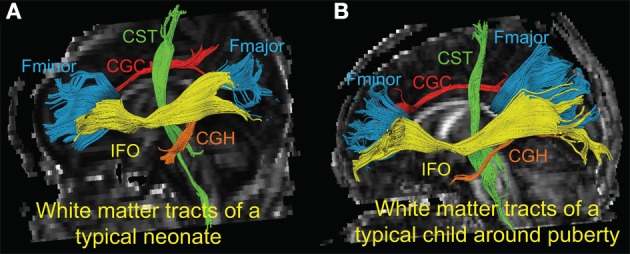
**3-D visualization of the traced WM tracts overlaid on mid-sagittal slice of the FA image of a typical neonate (A) and a typical child around puberty (B)**. Different colors represent different tracts traced for both subjects. CGC_L/R, CGH_L/R, CST_L/R, FMajor/FMinor and IFO_L/R are painted by red, orange, green, blue, and yellow color, respectively.

**Figure 2:**
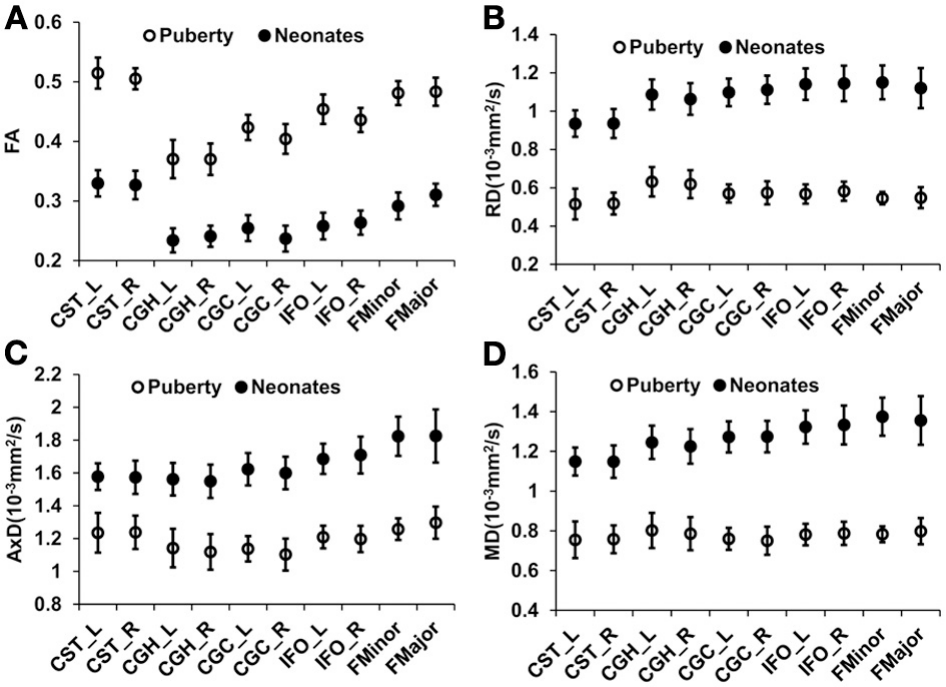
**Mean and standard deviation of tract-level FA (A), RD (B), AxD (C), and MD (D) measurements for 26 neonates and 28 children around puberty for all the 10 WM tracts**. Error bars indicate standard deviations across all the subjects at the same age group.

Figure 1 shows the three-dimensional (3D) visualization of the traced 10 major WM tracts for a typical neonate and a typical child around puberty. These 10 major tracts, namely CST_L, CST_R, IFO_L, IFO_R, CGC_L, CGC_R, CGH_L, CGH_R, FMajor and FMinor, cover projection, limbic, association and commissural tract groups involved in distinct brain functions. The microstructural changes from neonates to children at puberty and measured with FA, MD, AxD, and RD are shown in Figure 2. For all 10 major WM tracts, FA values are higher in the child group than those of the neonate group while MD, AxD, and RD values of the child group are less. FA of CST_L and CST_R of both age groups are highest among all tracts, followed by FMajor and FMinor. RD of CST_L and CST_R of both age groups are lowest among all tracts, indicating better myelination of CST compared to all other tracts. MD and AxD of FMajor and FMinor are highest among all tracts for child group. The values of these DTI-derived metrics of all tracts for the two age groups are shown in Supplemental Table 2.

### Enhanced inter-tract correlation from neonates to children around puberty

**Figure 3:**
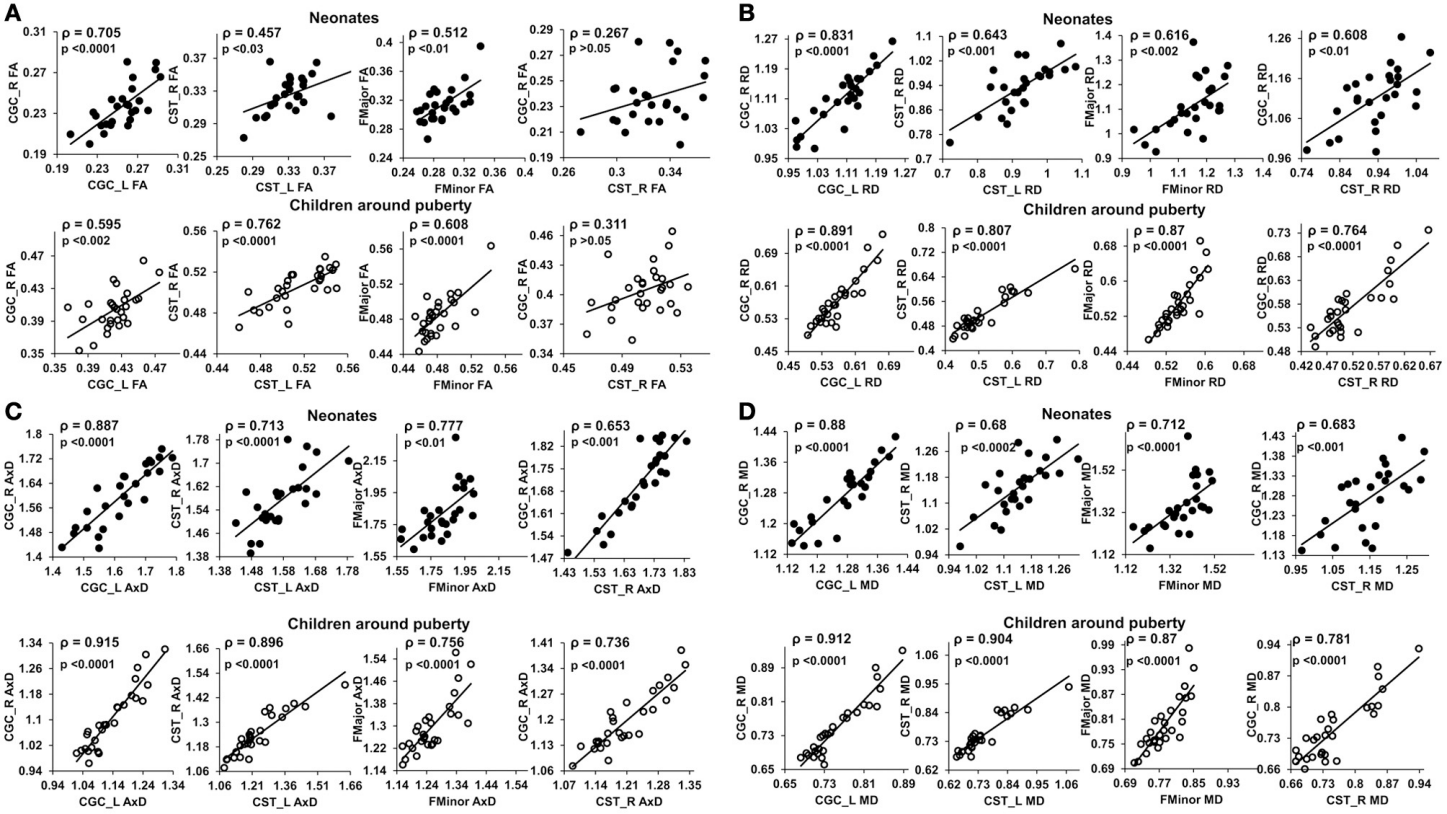
**Scatterplots of FA (A), RD (B), AxD (C) and MD (D) values from 2 homologous and 2 non-homologous tracts**. The 2 homologous pairs and FMajor/FMinor represent tract pairs of three tract groups for both age groups. Top panel shows the inter-tract scatter plots for the neonates and bottom panel shows those for the children around puberty. Each dot in all the plots represents the data from an individual subject in that group. ρ is the Spearman's rank correlation coefficient of the tract pair while *p*-value shows the statistical confidence of the inter-tract correlation strength.

Figure 3 shows the scatterplot of the FA (Figure 3A), RD (Figure 3B), AxD (Figure 3C), and MD (Figure 3D) values for 2 pairs of homologous tracts and 2 pairs of non-homologous tracts, CGC_L vs. CGC_R, CST_L vs. CST_R, FMajor vs. FMinor, and CST-R vs. CGC-R. The 2 homologous tracts and FMajor/FMinor represent tract pairs of the three tract groups. Significant correlations (*p* < 0.05) can be observed for these 4 pairs of tracts in both age groups.

**Figure 4:**
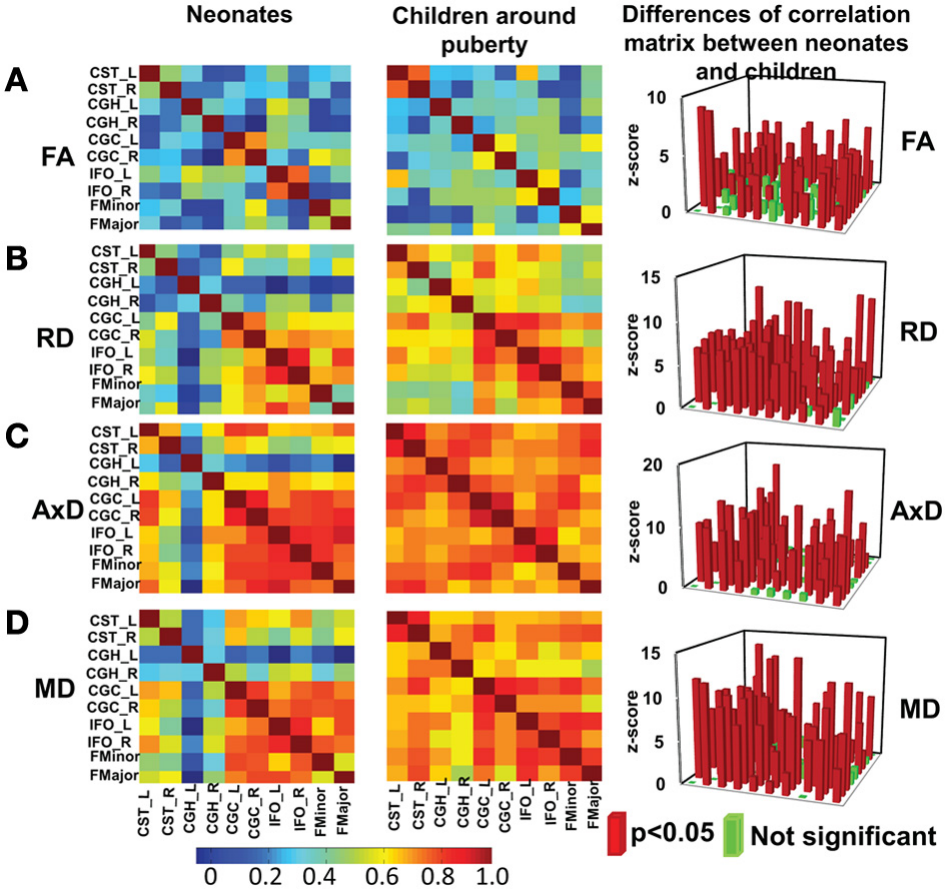
**Heat maps of the inter-tract correlation matrices obtained from tract-level FA (A), RD (B), AxD (C) and MD (D) measurements of both age groups**. The left and middle panels show the inter-tract correlation matrices for neonates and children around puberty, respectively. The right panel shows the *z*-scores of the changes in correlation strength between the two age groups. In the *z*-score plots, the entries with significant (*p* < 0.05) changes in inter-tract correlation strengths are shown in red color while entries with non-significant (*p* > 0.05) change in inter-tract correlation strengths are shown in green color. Color bar encoding the correlation strengths in the left and middle columns is also shown.

Significant differences (*p* < 0.05, Bonferroni-corrected) were observed for tests of all inter-tract correlation matrices of both groups against identity matrix or matrix with equal non-diagonal entries. The inter-tract correlation matrices of neonate and child group and the differences of these correlation matrices for FA, RD, AxD and MD are shown in Figures 4A–D, respectively. General stronger inter-tract correlations can be observed in the child group compared to those in the neonate group for all DTI-derived metrics, represented by the warmer colors in correlation matrices of child group. The statistics with *z*-scores (right panel of Figure 4) show that 64.4% (29/45), 84.4% (38/45), 73.3% (33/45), and 73.3% (33/45) of independent entries in the correlation matrix of child group are significantly higher than the corresponding entries of neonate group for FA, RD, AxD and MD, respectively. The correlation matrix from RD shows highest percentage changes (84.4%) among correlation matrices from all DTI-derived metrics, indicating more widespread enhanced inter-tract correlations with RD measurements. Note that the denominator 45 above indicates the number of all independent entries in the correlation matrix. The absolute values of correlation coefficients for AxD and MD, represented by the warmer colors in Figures 4C,D, are higher in both neonate and child group than those for FA (Figure 4A) or RD (Figure 4B). Much smaller percentages of independent entries of the correlation matrices are associated with the situation where correlation coefficients are significantly higher in the neonates than the children. Specifically, these percentages are 8.9% (4/45, namely CGC_R vs. FMinor, CGC_R vs. FMajor, CGH_L vs. IFO_L and IFO_L vs. IFO_R), 6.7% (3/45, namely CGC_R vs. FMinor, CGC_R vs. FMajor and IFO_L vs. FMajor), 4.4% (2/45, namely CGC_R vs. FMinor and CGC_R vs. FMajor) and 4.4% (2/45, namely CGC_R vs. IFO_R and CGC_R vs. FMajor) for FA, RD, AxD and MD, respectively. The inter-tract correlation coefficient values based on FA, RD, AxD and MD measurements for both age groups are shown in Supplemental Table 3.

### Reshuffled inter-tract correlation patterns from neonates to children around puberty

**Figure 5:**
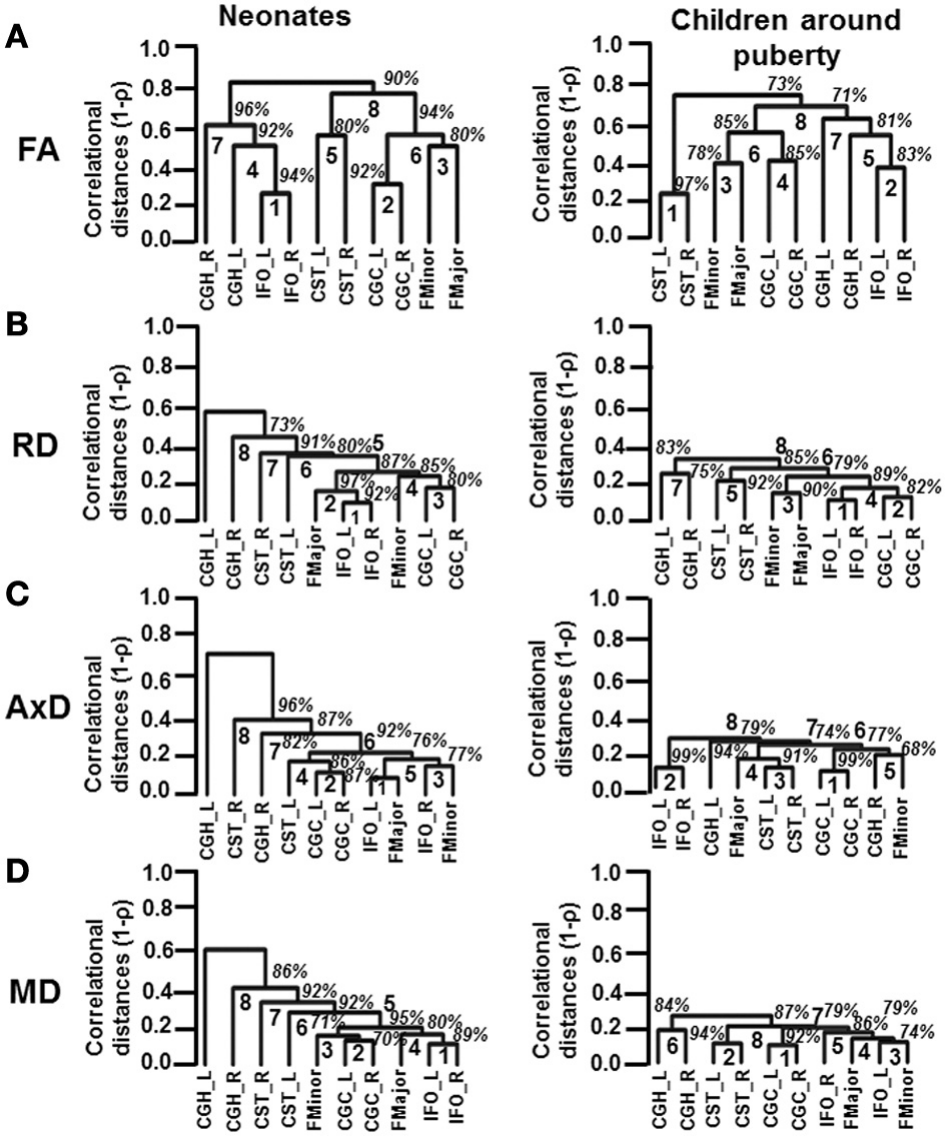
**Dendrograms depicting the hierarchical clustering pattern obtained from tract-level FA (A), RD (B), AxD (C) and MD (D) measurements for both age groups**. The left and right panels show the clustering pattern for neonates and children around puberty, respectively. The ranks of the clustering are shown in bold. The confidence intervals for the clustering (the percentage values) are shown in italics.

The inter-tract correlations based on each of DTI-derived metrics are reshuffled from neonates to children at puberty. These reshuffled inter-tract correlation patterns can be appreciated from dendrograms based on FA, RD, AxD, and MD measurements in Figures 5A–D, respectively. In general, the pairing of homologous tracts is more prominent for child group compared to that of the neonate group based on measurements of all DTI-derived metrics. The reshuffling leading to a more organized pairing among WM tracts is most prominent with the dendrograms based on tract-level RD measurements (Figure 5B). For inter-tract correlation based on FA measurement (Figure 5A), the tract pairs of CST_L/R becomes clear in the dendrogram of child group, while this pair is not as apparent in neonate group. The IFO_L/R pair is prominent for dendrograms of both neonate and child group based on FA measurements (Figure 5A). For dendrograms based on RD measurements (Figure 5B), it is clear that all homologous tracts and FMajor/FMinor get paired for child group while the correlation patterns are more random for neonate group. For dendrograms based on AxD (Figure 5C) or MD (Figure 5D) measurements, stronger and more clusters of the homologous tracts can be observed. The clustering pattern obtained from MD (Figure 5D) is similar to that obtained from RD (Figure 5B). However, the homologous tracts of child group are not well paired in MD-based dendrogram (Figure 5D), compared to those in RD-based dendrogram (Figure 5B).

## Discussion

In this study, dynamics of inter-tract correlations from birth to onset of adolescence was investigated with DTI-based tract-level microstructural measurements from 10 major WM tracts, CST_L, CST_R, IFO_L, IFO_R, CGC_L, CGC_R, CGH_L, CGH_R, FMajor, and FMinor. Higher WM tract integrity, reflected by higher FA, lower MD, AxD, and RD, were found for the corresponding tracts from neonates to children around puberty. It is clear that even at birth, nearly all major WM tracts demonstrate similar morphology as those in children around puberty. Significant correlations of homologous tracts are shown for both neonate and child groups. The comparisons of the inter-tract correlation matrices between the neonate and child group indicated that stronger inter-tract correlations are established during development. Using data-driven hierarchical clustering algorithm with no *a priori* information, we were able to reveal that the linkage patterns of the major tracts differ between the two age groups. Specifically, homologous tracts involved in similar brain functions tend to cluster together for children around puberty especially with tract-level RD measurements. Such clustering patterns of homologous tracts become more prominent from birth to puberty. These changes of inter-tract correlations between neonates and children around puberty suggest inhomogeneous but organized axonal development which causes the reshuffled inter-tract correlation pattern while keeping homologous tracts tightly correlated. To the best of our knowledge, this is the first study investigating dynamics of inter-tract correlations with DTI-based microstructural measurements during early human brain development.

### Heterogeneous WM growth

The WM development is heterogeneous among different tract groups, but more homogeneous among homologous tracts. The heterogeneity among different tract groups includes heterogeneous tract-level measurements of DTI-derived metrics at each time point and heterogeneous changes of these DTI-based tract-level measurements from birth to puberty. The general FA increase and general MD, AxD, and RD decrease for all WM tracts in early brain development shown in Figure 2 are consistent with the previous findings (e.g., Barnea-Goraly et al., 2005; Snook et al., 2005; Eluvathingal et al., 2007; Dubois et al., 2008; Lebel et al., 2008; Gao et al., 2009; Giorgio et al., 2010; Schmithorst and Yuan, 2010; Tamnes et al., 2010; Westlye et al., 2010). The heterogeneity among different tract groups is most prominent with FA measurements for children around puberty (Figure 2). From Figure 2A, it is clear that FA of CST-L/R (project tract group) is highest in children around puberty, followed by FMajor/FMinor (commissural tract group), IFO-L/R (association tract group) and CGC/H_L/R (limbic tract group). This order of tract group FA measurements is preserved back at birth, but the differences of FA among the tract groups are smaller (Figure 2A) for neonatal brains. The heterogeneous changes of tract-level DTI metrics in Figure 2 may cause the reshuffling of inter-tract correlation patterns. Quite similar FA values (Figure 2A) as well as MD, AxD, and RD values (Figures 2B,D) of the homologous tracts can be found for both age groups.

### Strengthened and reshuffled inter-tract correlation during development

At birth, significant correlations can be observed based on microstructural measurements of homologous tracts, as shown in upper panels of Figures 3A,D. From Figure 4, the general inter-tract correlations from birth to puberty are clearly stronger for all four DTI-derived microstructural measurements. With FA measurement as an example, each entry of correlation matrix in Figure 4A can be expanded to the correlation scatterplots such as those shown in panels of Figure 3A. Our results also indicated that significantly increased inter-tract correlations are more widespread with correlation coefficients based on RD measurements. For both age groups, correlation coefficients are highest based on AxD measurements (Figure 4C) and lowest based on FA measurements (FA). This pattern is especially clear for children around puberty. The overall higher inter-tract correlation coefficients based on AxD measurements suggest that axonal integrity is coherent among the WM tracts within an individual child's brain but varies among different children. To help understand this finding, we could assume a situation when each of the children around puberty has identical AxD for all 10 major WM tracts and the AxD values vary among these children. Under such situation, all inter-tract correlation coefficients based on AxD would be the perfect value 1. The relatively low inter-tract correlation coefficients based on FA measurements may be caused by heterogeneity of FA values among the WM tracts within each individual subject's brain. Larger variability of FA values among WM tracts can also be observed in Figure 2A.

From Figure 5A, the FA-based dendrograms show that several homologous tracts are clustered together even for neonates. The left and right IFO are clustered with rank 1 for dendrograms based on tract-level FA of neonatal brains. IFO is thought to play a role in integrating the information from auditory and visual cortices to the prefrontal cortex (e.g., Martino et al., 2010). Resting-state fMRI studies have shown consistent pattern of activation in auditory and visual networks in neonates (Fransson et al., 2007; Doria et al., 2010). It is noteworthy that in the resting-state fMRI studies, the connectivity was also identified through correlation of brain-oxygen-level-dependent (BOLD) signal fluctuations in the homologous brain regions. The strong correlations of BOLD signal time courses in visual and auditory networks between left and right hemisphere in neonatal brains may be related to tight cluster of IFO-L and IFO-R involved in these brain functions. Both neonatal and child dendrograms based on FA measurements (Figure 5A) have two more separate clusters of projection tracts (CST) and limbic tracts (CGC). All these clusters can also be found in adult brains (Wahl et al., 2010). Figure 5B demonstrates one of the most compelling findings with dendrograms based on RD measurements. Although two pairs of homologous WM tracts are clustered together with rank 1 (IFO_L/R) and rank 3 (CGC_L/R), the other two pairs of homologous tracts and FMajor/FMinor are spread all over the dendrogram for neonatal brains (Figure 5B). It is striking that all 4 pairs of homologous tracts and FMajor/FMinor are tightly clustered together for children around puberty (Figure 5B). RD is closely related to myelination of WM tracts (Song et al., 2002). We should be careful to associate the RD values with myelination due to crossing-fiber and pathological situations (Wheeler-Kingshott and Cercignani, 2009). Nevertheless, during normal brain development with no implication of pathology and with the assumption that tract-level RD measurement for major WM tracts is much less affected by crossing fiber compared to voxelwise RD measurement, tract-level RD is still considered as an important index reflecting the degree of myelination (e.g., Snook et al., 2005; Eluvathingal et al., 2007; Gao et al., 2009; Tamnes et al., 2010; Westlye et al., 2010). The results in Figure 5B suggest that organized myelination from birth to puberty plays an important role to reshuffle the inter-tract correlations and result in clustered homologous tracts and clustered functionally similar tracts. The dendrogram patterns based on AxD and MD measurements are different than those based on FA or RD measurements. Unlike RD-based dendrogram (Figure 5B), neither of the dendrograms based on AxD or MD measurements for children around puberty shows well organized clusters of all 4 pairs of homologous tracts and FMajor/FMinor (Figures 5C,D). Previous studies (Mukherjee et al., 2002; Snook et al., 2005; Gao et al., 2009) found that the measurements of RD decrease dramatically with relatively little changes in AxD of the major WM tracts during brain development. Our results demonstrated in Figure 2 also indicate smaller and more homogeneous changes of AxD from birth to puberty compared to those of RD. These AxD change features may explain why relatively disorganized AxD-based dendrograms remain for children around puberty. From Equation (1), MD is the linear combination of AxD and RD. The relatively disorganized dendrograms based on MD measurement for child group could also be originated from smaller and more homogeneous changes of AxD measurements during development. Nevertheless, with the brain development, there are still trends for homologous and functionally similar tracts to cluster together in dendrograms based on AxD and MD in Figures 5C,D.

### Possible mechanisms of the inter-tract correlation changes from birth to onset of adolescence

This study on dynamics of inter-tract correlations from birth to onset of adolescence provides unique insight on the well-organized cerebral WM development. The development of human cerebral WM tracts is characterized with enhanced myelination and axonal integrity. From the results in this study, inhomogeneous RD decreases among the tracts take place during development. More (84.4% of all independent correlation coefficients) inter-tract correlations become stronger with RD measurements, compared to those with any other DTI metric measurements. In addition, the dendrograms based on RD (Figure 5B) demonstrate that all 4 pairs of homologous tracts and FMajor/FMinor are tightly clustered at puberty while only 2 pairs of homologous tracts are clustered at birth. The cerebral WM at birth is likely to be in a relatively random and disorganized status. Due to close relationship of RD with the myelination of WM tract, these results suggest that inhomogeneous enhancement of myelination rather than strengthening of axonal integrity plays a key role in reshaping the WM configuration during development. Both genes and experiences could also play a role to adjust WM microstructures so that the homologous WM tracts reach to a coherent status to meet the needs of certain brain functions by the time of onset of adolescence. Although it is not known by what mechanism inhomogeneous myelination is modulated during WM maturation, our results suggest that the myelination process is precisely controlled so that all 4 pairs of homologous tracts and FMajor/FMinor are clustered together around puberty (Figure 5B).

### Limitations of this study and future directions

There are several issues which may affect the results in this study. Five pairs of major WM tracts were chosen for this study due to the fact that only these five pairs of major WM tracts could be reproducibly traced with neonate DTI. The numbers of participated subjects, 26 for neonates and 28 for children, just exceeds 25 which is needed for correlation analysis of five pairs of WM tracts. Higher sample numbers could increase the confidence level in analysis of hierarchical clustering. Corrections for multiple comparisons were only performed on testing the correlation matrices against identity matrix and matrix with equal non-diagonal elements. No correction was performed on the hierarchical clustering results due to lack of any known methods to perform such a correction on dendrograms. The accuracy of measuring tract-level DTI-derived metrics plays a key role in inter-tract correlations. This accuracy is affected by three major factors. They are the crossing-fiber factor, SNR of the data and partial volume effects. Both the tractography and the DTI-derived measurements are biased at crossing-fiber regions (Wheeler-Kingshott and Cercignani, 2009). With single tensor model and tractography method of fiber assignment by continuous tracking (FACT) (Mori et al., 1999), it is apparent that the tracing method adopted in this study cannot resolve the crossing-fiber issue, resulting in imperfect binary mask of the traced tracts for tract-level measurements of DTI metrics. Nevertheless, the tracing protocol (Wakana et al., 2007) based on FACT tractography captures the core of the major WM tracts and is still widely used in the field. We conducted two repetitions of diffusion MRI and the SNR is sufficient for data acquisition with 3T magnet. With diffusion imaging resolution 2 × 2 × 2 mm^3^ for neonates and 2 × 2 × 2.2 mm^3^ for children around puberty, the partial volume effects are inevitable. However, it seems the effects of imperfect WM fiber tracing offsets the partial volume effects for obtaining accurate tract-level DTI metrics, in that the tracing algorithm adopted in this study cannot trace the small branches of the fibers where the problem of partial volume effects is most prominent. Ranks of the DTI measurements, instead of measured metrics themselves, were used for correlation pattern analysis. Despite possibly different levels of biases of the DTI metric measurements caused by partial volume effects due to different head sizes between neonates and children, the similar shifts of measurements in the same age group could have minimum effects on the rank of metric measurements and therefore minimum effects on the Spearman's rank correlation patterns. Although same types of scanners were used in this study, systematic differences of the scanners may affect the DTI analysis results. To make sure that the effects of systematic differences caused by two different scanners are minimal, a healthy young subject (“*in vivo* human phantom”) was scanned in both scanners used in this study and with the same DTI sequence. The quantitative comparisons were also conducted. Quantitative DTI measurement differences caused by scanner difference were tested to be within the range of variability of scanning the same subject twice with one scanner (Saxena et al., 2012). With the same type of scanners used in this study and rigorous quality control of both scanners, the effects of scanner differences on the presented results in this study are thus negligible. The group of children around puberty included both pre-puberty and post-puberty subjects. The age difference between the two groups, newborns and children around puberty, is much larger than the age difference within the group of around puberty. Therefore, we hypothesized that the intra-group WM developmental heterogeneity for the children group exists, but is not big enough to affect the inter-group results presented in this study. This has been tested and proved by Supplemental Figure 1. In Supplemental Figure 1, we separated the children around puberty into two subgroups, pre-puberty (9.5–12 years) and post-puberty (12–15 years). With reduced sample number for each subgroup, we could conduct the correlation analysis for 6 tracts (3 pairs of homologous tracts). It is shown in Supplemental Figure 1 that homologous tracts are still clustered together in both subgroups, like the cluster patterns shown in the right column of Figure 5. Moreover, the linkage patterns are very similar between the two subgroups, with slight dendrogram rank change.

In the future, several improvements can be made to address the issues affecting accuracy of measuring tract-level DTI metrics. The tracking methods (e.g., Tournier et al., 2004; Behrens et al., 2007) which are capable of resolving the crossing-fiber issue can be adopted. Although there is no general consensus on which parameters can replace these four DTI-derived metrics and better characterize the WM microstructure, there have been a few metrics such as general fractional anisotropy (GFA) (Tuch, 2004; Fritzsche et al., 2010; Zhan et al., 2010), generalized anisotropy (GA) (Ozarslan et al., 2005), mode of anisotropy (Ennis and Kindlmann, 2006; Douaud et al., 2011) and fractions (Hosey et al., 2008; Jbabdi et al., 2010) which are less sensitive to crossing-fiber problem. In addition, tract-based spatial statistics (TBSS) from FSL (http://www.fmrib.ox.ac.uk/fsl) (Smith et al., 2006) can be used to alleviate the partial volume effects, as shown in the study of FA correlations in adults (Li et al., 2012).

## Conclusion

In conclusion, inter-tract correlation changes during development from birth to onset of adolescence were investigated with tract-level FA, RD, AxD, and MD measurements. Stronger and enhanced microstructural inter-tract correlations were found during development. The linkage patterns of the major tracts also differ with the dendrograms of two age groups due to brain development. These changes of microstructural correlations from birth to puberty suggest inhomogeneous but organized myelination processes which cause the reshuffled inter-tract correlation pattern and make homologous tracts tightly clustered. Especially RD-based dendrograms reveal that all 4 pairs of homologous tracts and FMajor/FMinor investigated in this study are tightly clustered for children around puberty while only 2 out of these 5 pairs of tracts are clustered at birth, indicating important role of myelination to reshape the WM configuration. It opens a new window to study WM tract development and can be potentially used to investigate atypical brain development due to neurological or psychiatric disorders.

## Conflict of interest statement

The authors declare that the research was conducted in the absence of any commercial or financial relationships that could be construed as a potential conflict of interest.

## Supplementary material

Supplementary Figure 1 **Dendrograms depicting the hierarchical clustering pattern obtained from tract-level FA (A), RD (B), AxD (C) and MD (D) measurements for two subgroups of children around puberty**. Dendrograms on the left column are for the subgroup of children younger than 12-years-old (9.5–12years); those on the right column are for the subgroup of children older than 12-year-old (12–15years).
