# Supplementary material for: Differences of inter-tract correlations between neonates and children around puberty: a study based on microstructural measurements with DTI
Source: Front Hum Neurosci. 2013 Oct 29;7:721. doi: 10.3389/fnhum.2013.00721 (PMC3810597; doi:10.3389/fnhum.2013.00721)
Supplement: Supplementary Figure 1 — Dendrograms depicting the hierarchical clustering pattern obtained from tract-level FA (A), RD (B), AxD (C) and MD (D) measurements for two subgroups of children around puberty. Dendrograms on the left column are for the subgroup of children younger than 12-years-old (9.5–12years); those on the right column are for the subgroup of children older than 12-year-old (12–15years). [file Presentation1.PDF]

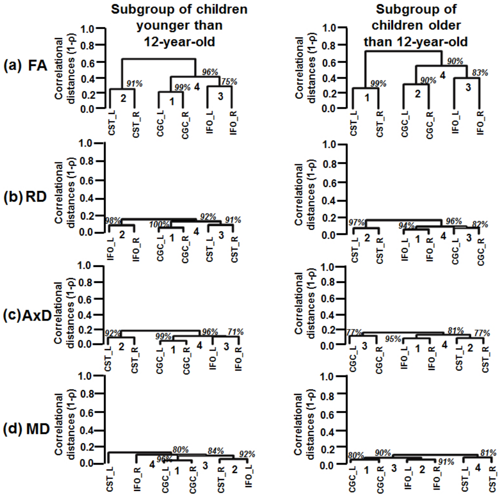

Subgroup of children younger than 12-year-old
Subgroup of children older than 12-year-old
(a) FA
(b) RD
(c) AxD
(d) MD
Correlational distances (1-ρ)
CST_L
CST_R
CGC_L
CGC_R
IFO_L
IFO_R
