# Supplementary material for: Differences of inter-tract correlations between neonates and children around puberty: a study based on microstructural measurements with DTI
Source: Front Hum Neurosci. 2013 Oct 29;7:721. doi: 10.3389/fnhum.2013.00721 (PMC3810597; doi:10.3389/fnhum.2013.00721)
Supplement: Supplementary file 2 [file DataSheet1.DOCX]

**Supplemental Table 1a: Mean coefficients of variation (% CV) of FA, RD, AxD and MD measurements for neonates and children around puberty. 3 subjects in each group were randomly selected to compute CV. Each tract was traced 3 times for data from each subject.**

|  | **FA** | | **RD** | | **AxD** | | **MD** | |
| --- | --- | --- | --- | --- | --- | --- | --- | --- |
|  | ***Neonates*** | ***Children around puberty*** | ***Neonates*** | ***Children around puberty*** | ***Neonates*** | ***Children around puberty*** | ***Neonates*** | ***Children around puberty*** |
| **CST_L (%)** | 0.2 | 0.2 | 0.1 | 0.1 | 0.06 | 0.2 | 0.05 | 0.05 |
| **CST_R (%)** | 0.2 | 0.1 | 0.1 | 0.2 | 0.1 | 0.1 | 0.1 | 0.1 |
| **CGH_L (%)** | 0 | 0.1 | 0 | 0.03 | 0 | 0.06 | 0 | 0.03 |
| **CGH_R (%)** | 0.02 | 0.1 | 0.001 | 0.02 | 0.02 | 0.08 | 0.008 | 0.05 |
| **CGC_L (%)** | 0 | 0.07 | 0 | 0.05 | 0 | 0.05 | 0 | 0.03 |
| **CGC_R (%)** | 0 | 0.06 | 0 | 0.1 | 0 | 0.06 | 0 | 0.08 |
| **IFO_L (%)** | 0.01 | 0.03 | 0.002 | 0.03 | 0.001 | 0.03 | 0.001 | 0.01 |
| **IFO_R (%)** | 0 | 0.07 | 0 | 0.06 | 0 | 0.04 | 0 | 0.04 |
| **FMinor (%)** | 0.2 | 0.05 | 0.2 | 0.02 | 0.2 | 0.04 | 0.2 | 0.01 |
| **FMajor (%)** | 0.3 | 0.03 | 0.03 | 0.01 | 0.1 | 0.02 | 0.05 | 0.01 |
| **Average (%)** | 0.09 | 0.08 | 0.04 | 0.06 | 0.05 | 0.07 | 0.04 | 0.04 |

**Supplemental Table 1b: Mean Kappa (κ) for neonates and children around puberty. 3 subjects in each group were randomly selected to compute κ. Each tract was traced 3 times for data from each subject.**

|  | **Mean Kappa (κ)** | |
| --- | --- | --- |
|  | ***Neonates*** | ***Children around puberty*** |
| **CST_L** | 0.986 | 0.962 |
| **CST_R** | 0.97 | 0.979 |
| **CGH_L** | 0.99 | 0.987 |
| **CGH_R** | 0.989 | 0.987 |
| **CGC_L** | 0.99 | 0.984 |
| **CGC_R** | 0.989 | 0.979 |
| **IFO_L** | 0.995 | 0.986 |
| **IFO_R** | 0.996 | 0.982 |
| **FMinor** | 0.956 | 0.997 |
| **FMajor** | 0.975 | 0.998 |
| **Average** | 0.984 | 0.984 |
