# Supplementary material for: Differences of inter-tract correlations between neonates and children around puberty: a study based on microstructural measurements with DTI
Source: Front Hum Neurosci. 2013 Oct 29;7:721. doi: 10.3389/fnhum.2013.00721 (PMC3810597; doi:10.3389/fnhum.2013.00721)
Supplement: Supplementary file 3 [file DataSheet2.DOCX]

**Supplemental Table 2: Mean tract-level FA, RD, AxD and MD measurements and their standard deviations for neonates and children around puberty.**

|  | **FA** | | **RD (10^-3^mm^2^/s)** | | **AxD (10^-3^mm^2^/s)** | | **MD (10^-3^mm^2^/s)** | |
| --- | --- | --- | --- | --- | --- | --- | --- | --- |
|  | ***Neonates*** | ***Children around puberty*** | ***Neonates*** | ***Children around puberty*** | ***Neonates*** | ***Children around puberty*** | ***Neonates*** | ***Children around puberty*** |
|  |  |  |  |  |  |  |  |  |
| **CST left** | 0.33±0.022 | 0.514±0.026 | 0.935±0.069 | 0.515±0.08 | 1.577±0.081 | 1.235±0.121 | 1.149±0.07 | 0.755±0.092 |
| **CST right** | 0.327±0.024 | 0.505±0.018 | 0.935±0.076 | 0.518±0.057 | 1.573±0.101 | 1.238±0.102 | 1.148±0.082 | 0.758±0.07 |
| **CGH left** | 0.234±0.02 | 0.37±0.032 | 1.087±0.079 | 0.631±0.077 | 1.562±0.099 | 1.142±0.117 | 1.245±0.084 | 0.802±0.088 |
| **CGH right** | 0.241±0.018 | 0.37±0.026 | 1.063±0.082 | 0.619±0.073 | 1.549±0.102 | 1.119±0.108 | 1.225±0.087 | 0.786±0.083 |
| **CGC left** | 0.254±0.022 | 0.423±0.021 | 1.098±0.072 | 0.57±0.047 | 1.622±0.099 | 1.139±0.077 | 1.273±0.078 | 0.759±0.055 |
| **CGC right** | 0.237±0.022 | 0.404±0.025 | 1.112±0.074 | 0.574±0.06 | 1.6±0.099 | 1.103±0.098 | 1.274±0.079 | 0.75±0.071 |
| **IFO left** | 0.258±0.022 | 0.454±0.025 | 1.141±0.082 | 0.567±0.05 | 1.686±0.092 | 1.209±0.069 | 1.322±0.084 | 0.781±0.054 |
| **IFO right** | 0.264±0.02 | 0.436±0.02 | 1.145±0.093 | 0.582±0.05 | 1.709±0.112 | 1.197±0.08 | 1.333±0.098 | 0.787±0.058 |
| **FMinor** | 0.292±0.023 | 0.481±0.02 | 1.15±0.088 | 0.546±0.032 | 1.823±0.119 | 1.257±0.066 | 1.374±0.095 | 0.783±0.04 |
| **FMajor** | 0.31±0.019 | 0.483±0.024 | 1.121±0.105 | 0.548±0.055 | 1.825±0.162 | 1.297±0.098 | 1.355±0.122 | 0.798±0.066 |
