# Supplementary material for: Differences of inter-tract correlations between neonates and children around puberty: a study based on microstructural measurements with DTI
Source: Front Hum Neurosci. 2013 Oct 29;7:721. doi: 10.3389/fnhum.2013.00721 (PMC3810597; doi:10.3389/fnhum.2013.00721)
Supplement: Supplementary file 4 [file DataSheet3.DOCX]

**Supplemental Table 3a: Spearman’s inter-tract correlation coefficient (ρ) matrix based on FA measurements.**

| **Neonates** | | | | | | | | | | |
| --- | --- | --- | --- | --- | --- | --- | --- | --- | --- | --- |
|  | **CST_L** | **CST_R** | **CGH_L** | **CGH_R** | **CGC_L** | **CGC_R** | **IFO_L** | **IFO_R** | **FMinor** | **FMajor** |
| **CST_L** | 1 |  |  |  |  |  |  |  |  |  |
| **CST_R** | 0.457 | 1 |  |  |  |  |  |  |  |  |
| **CGH_L** | 0.318 | 0.181 | 1 |  |  |  |  |  |  |  |
| **CGH_R** | 0.05 | 0.186 | 0.372 | 1 |  |  |  |  |  |  |
| **CGC_L** | 0.088 | 0.314 | 0.294 | 0.136 | 1 |  |  |  |  |  |
| **CGC_R** | 0.252 | 0.267 | 0.144 | -0.074 | 0.705 | 1 |  |  |  |  |
| **IFO_L** | 0.44 | 0.29 | 0.556 | 0.454 | 0.47 | 0.3 | 1 |  |  |  |
| **IFO_R** | 0.19 | 0.143 | 0.463 | 0.388 | 0.358 | 0.167 | 0.752 | 1 |  |  |
| **FMinor** | 0.278 | 0.331 | 0 | 0 | 0.352 | 0.587 | 0.196 | 0.036 | 1 |  |
| **FMajor** | 0.324 | 0.093 | 0.182 | 0.026 | 0.378 | 0.498 | 0.383 | 0.086 | 0.512 | 1 |

| **Children around puberty** | | | | | | | | | | |
| --- | --- | --- | --- | --- | --- | --- | --- | --- | --- | --- |
|  | **CST_L** | **CST_R** | **CGH_L** | **CGH_R** | **CGC_L** | **CGC_R** | **IFO_L** | **IFO_R** | **FMinor** | **FMajor** |
| **CST_L** | 1 |  |  |  |  |  |  |  |  |  |
| **CST_R** | 0.762 | 1 |  |  |  |  |  |  |  |  |
| **CGH_L** | 0.267 | 0.244 | 1 |  |  |  |  |  |  |  |
| **CGH_R** | 0.256 | 0.045 | 0.347 | 1 |  |  |  |  |  |  |
| **CGC_L** | 0.301 | 0.363 | 0.390 | 0.241 | 1 |  |  |  |  |  |
| **CGC_R** | 0.143 | 0.311 | 0.369 | 0.385 | 0.595 | 1 |  |  |  |  |
| **IFO_L** | 0.655 | 0.395 | 0.344 | 0.467 | 0.419 | 0.351 | 1 |  |  |  |
| **IFO_R** | 0.331 | 0.033 | 0.457 | 0.465 | 0.469 | 0.494 | 0.631 | 1 |  |  |
| **FMinor** | 0.006 | 0.055 | 0.152 | 0.041 | 0.46 | 0.39 | 0.086 | 0.274 | 1 |  |
| **FMajor** | 0.414 | 0.397 | 0.466 | 0.221 | 0.595 | 0.365 | 0.335 | 0.427 | 0.608 | 1 |

**Supplemental Table 3b: Spearman’s inter-tract correlation coefficient (ρ) matrix based on RD measurements.**

| **Neonates** | | | | | | | | | | |
| --- | --- | --- | --- | --- | --- | --- | --- | --- | --- | --- |
|  | **CST_L** | **CST_R** | **CGH_L** | **CGH_R** | **CGC_L** | **CGC_R** | **IFO_L** | **IFO_R** | **FMinor** | **FMajor** |
| **CST_L** | 1 |  |  |  |  |  |  |  |  |  |
| **CST_R** | 0.643 | 1 |  |  |  |  |  |  |  |  |
| **CGH_L** | 0.508 | 0.432 | 1 |  |  |  |  |  |  |  |
| **CGH_R** | 0.406 | 0.495 | 0.558 | 1 |  |  |  |  |  |  |
| **CGC_L** | 0.631 | 0.748 | 0.454 | 0.628 | 1 |  |  |  |  |  |
| **CGC_R** | 0.724 | 0.608 | 0.452 | 0.508 | 0.831 | 1 |  |  |  |  |
| **IFO_L** | 0.682 | 0.595 | 0.323 | 0.668 | 0.690 | 0.796 | 1 |  |  |  |
| **IFO_R** | 0.748 | 0.693 | 0.469 | 0.680 | 0.764 | 0.824 | 0.909 | 1 |  |  |
| **FMinor** | 0.595 | 0.551 | 0.357 | 0.486 | 0.738 | 0.802 | 0.716 | 0.79 | 1 |  |
| **FMajor** | 0.619 | 0.726 | 0.368 | 0.666 | 0.744 | 0.795 | 0.878 | 0.818 | 0.616 | 1 |

| **Children around puberty** | | | | | | | | | | |
| --- | --- | --- | --- | --- | --- | --- | --- | --- | --- | --- |
|  | **CST_L** | **CST_R** | **CGH_L** | **CGH_R** | **CGC_L** | **CGC_R** | **IFO_L** | **IFO_R** | **FMinor** | **FMajor** |
| **CST_L** | 1 |  |  |  |  |  |  |  |  |  |
| **CST_R** | 0.807 | 1 |  |  |  |  |  |  |  |  |
| **CGH_L** | 0.743 | 0.639 | 1 |  |  |  |  |  |  |  |
| **CGH_R** | 0.692 | 0.737 | 0.771 | 1 |  |  |  |  |  |  |
| **CGC_L** | 0.815 | 0.861 | 0.72 | 0.694 | 1 |  |  |  |  |  |
| **CGC_R** | 0.76 | 0.764 | 0.745 | 0.762 | 0.891 | 1 |  |  |  |  |
| **IFO_L** | 0.828 | 0.738 | 0.78 | 0.712 | 0.894 | 0.837 | 1 |  |  |  |
| **IFO_R** | 0.784 | 0.707 | 0.697 | 0.723 | 0.846 | 0.799 | 0.906 | 1 |  |  |
| **FMinor** | 0.65 | 0.639 | 0.654 | 0.584 | 0.806 | 0.719 | 0.851 | 0.826 | 1 |  |
| **FMajor** | 0.669 | 0.712 | 0.668 | 0.588 | 0.823 | 0.678 | 0.795 | 0.787 | 0.87 | 1 |

**Supplemental Table 3c: Spearman’s inter-tract correlation coefficient (ρ) matrix based on AxD measurements.**

| **Neonates** | | | | | | | | | | |
| --- | --- | --- | --- | --- | --- | --- | --- | --- | --- | --- |
|  | **CST_L** | **CST_R** | **CGH_L** | **CGH_R** | **CGC_L** | **CGC_R** | **IFO_L** | **IFO_R** | **FMinor** | **FMajor** |
| **CST_L** | 1 |  |  |  |  |  |  |  |  |  |
| **CST_R** | 0.713 | 1 |  |  |  |  |  |  |  |  |
| **CGH_L** | 0.38 | 0.259 | 1 |  |  |  |  |  |  |  |
| **CGH_R** | 0.68 | 0.541 | 0.43 | 1 |  |  |  |  |  |  |
| **CGC_L** | 0.835 | 0.711 | 0.33 | 0.673 | 1 |  |  |  |  |  |
| **CGC_R** | 0.825 | 0.653 | 0.287 | 0.681 | 0.887 | 1 |  |  |  |  |
| **IFO_L** | 0.691 | 0.535 | 0.166 | 0.752 | 0.748 | 0.832 | 1 |  |  |  |
| **IFO_R** | 0.708 | 0.503 | 0.282 | 0.691 | 0.813 | 0.813 | 0.860 | 1 |  |  |
| **FMinor** | 0.712 | 0.639 | 0.32 | 0.656 | 0.828 | 0.828 | 0.848 | 0.854 | 1 |  |
| **FMajor** | 0.786 | 0.646 | 0.077 | 0.686 | 0.798 | 0.868 | 0.915 | 0.792 | 0.777 | 1 |

| **Children around puberty** | | | | | | | | | | |
| --- | --- | --- | --- | --- | --- | --- | --- | --- | --- | --- |
|  | **CST_L** | **CST_R** | **CGH_L** | **CGH_R** | **CGC_L** | **CGC_R** | **IFO_L** | **IFO_R** | **FMinor** | **FMajor** |
| **CST_L** | 1 |  |  |  |  |  |  |  |  |  |
| **CST_R** | 0.896 | 1 |  |  |  |  |  |  |  |  |
| **CGH_L** | 0.748 | 0.779 | 1 |  |  |  |  |  |  |  |
| **CGH_R** | 0.802 | 0.85 | 0.814 | 1 |  |  |  |  |  |  |
| **CGC_L** | 0.836 | 0.817 | 0.72 | 0.813 | 1 |  |  |  |  |  |
| **CGC_R** | 0.782 | 0.736 | 0.706 | 0.772 | 0.915 | 1 |  |  |  |  |
| **IFO_L** | 0.709 | 0.757 | 0.745 | 0.750 | 0.703 | 0.778 | 1 |  |  |  |
| **IFO_R** | 0.725 | 0.764 | 0.712 | 0.77 | 0.808 | 0.815 | 0.898 | 1 |  |  |
| **FMinor** | 0.791 | 0.798 | 0.765 | 0.83 | 0.857 | 0.764 | 0.675 | 0.733 | 1 |  |
| **FMajor** | 0.833 | 0.869 | 0.782 | 0.688 | 0.769 | 0.706 | 0.749 | 0.704 | 0.756 | 1 |

**Supplemental Table 3d: Spearman’s inter-tract correlation coefficient (ρ) matrix based on MD measurements.**

| **Neonates** | | | | | | | | | | |
| --- | --- | --- | --- | --- | --- | --- | --- | --- | --- | --- |
|  | **CST_L** | **CST_R** | **CGH_L** | **CGH_R** | **CGC_L** | **CGC_R** | **IFO_L** | **IFO_R** | **FMinor** | **FMajor** |
| **CST_L** | 1 |  |  |  |  |  |  |  |  |  |
| **CST_R** | 0.68 | 1 |  |  |  |  |  |  |  |  |
| **CGH_L** | 0.454 | 0.459 | 1 |  |  |  |  |  |  |  |
| **CGH_R** | 0.533 | 0.56 | 0.549 | 1 |  |  |  |  |  |  |
| **CGC_L** | 0.794 | 0.779 | 0.439 | 0.675 | 1 |  |  |  |  |  |
| **CGC_R** | 0.76 | 0.683 | 0.469 | 0.569 | 0.88 | 1 |  |  |  |  |
| **IFO_L** | 0.727 | 0.612 | 0.301 | 0.692 | 0.787 | 0.826 | 1 |  |  |  |
| **IFO_R** | 0.807 | 0.705 | 0.485 | 0.712 | 0.846 | 0.869 | 0.897 | 1 |  |  |
| **FMinor** | 0.648 | 0.628 | 0.378 | 0.56 | 0.861 | 0.849 | 0.765 | 0.848 | 1 |  |
| **FMajor** | 0.701 | 0.735 | 0.306 | 0.642 | 0.819 | 0.864 | 0.867 | 0.83 | 0.712 | 1 |

| **Children around puberty** | | | | | | | | | | |
| --- | --- | --- | --- | --- | --- | --- | --- | --- | --- | --- |
|  | **CST_L** | **CST_R** | **CGH_L** | **CGH_R** | **CGC_L** | **CGC_R** | **IFO_L** | **IFO_R** | **FMinor** | **FMajor** |
| **CST_L** | 1 |  |  |  |  |  |  |  |  |  |
| **CST_R** | 0.904 | 1 |  |  |  |  |  |  |  |  |
| **CGH_L** | 0.768 | 0.791 | 1 |  |  |  |  |  |  |  |
| **CGH_R** | 0.764 | 0.832 | 0.836 | 1 |  |  |  |  |  |  |
| **CGC_L** | 0.814 | 0.889 | 0.775 | 0.758 | 1 |  |  |  |  |  |
| **CGC_R** | 0.776 | 0.781 | 0.756 | 0.79 | 0.912 | 1 |  |  |  |  |
| **IFO_L** | 0.777 | 0.827 | 0.819 | 0.727 | 0.881 | 0.805 | 1 |  |  |  |
| **IFO_R** | 0.788 | 0.83 | 0.725 | 0.736 | 0.853 | 0.81 | 0.868 | 1 |  |  |
| **FMinor** | 0.812 | 0.856 | 0.799 | 0.732 | 0.886 | 0.808 | 0.897 | 0.878 | 1 |  |
| **FMajor** | 0.783 | 0.866 | 0.77 | 0.672 | 0.832 | 0.71 | 0.886 | 0.796 | 0.87 | 1 |
